# Spatial parcellations, spectral filtering, and connectivity measures in fMRI: Optimizing for discrimination

**DOI:** 10.1002/hbm.24381

**Published:** 2018-09-26

**Authors:** Roser Sala‐Llonch, Stephen M. Smith, Mark Woolrich, Eugene P. Duff

**Affiliations:** ^1^ Faculty of Medicine, Department of Biomedicine University of Barcelona Barcelona Spain; ^2^ Nuffield Department of Clinical Neurosciences, Functional Magnetic Resonance Imaging of the Brain Centre, Wellcome Centre for Integrative Neuroimaging University of Oxford Oxford United Kingdom; ^3^ Department of Psychiatry, Oxford University Centre for Human Brain Activity, Wellcome Centre for Integrative Neuroimaging University of Oxford Oxford United Kingdom; ^4^ Department of Paediatrics University of Oxford Oxford United Kingdom

**Keywords:** correlation, covariance, fMRI, functional connectivity, partial correlation, rfMRI

## Abstract

The analysis of Functional Connectivity (FC) is a key technique of fMRI, having been used to distinguish brain states and conditions. While many approaches to calculating FC are available, there have been few assessments of their differences, making it difficult to choose approaches, and compare results. Here, we assess the impact of methodological choices on discriminability, using a fully controlled data set of continuous active states involving basic visual and motor tasks, providing robust localized FC changes. We tested a range of anatomical and functional parcellations, including the AAL atlas, parcellations derived from the Human Connectome Project and Independent Component Analysis (ICA) of many dimensionalities. We measure amplitude, covariance, correlation, and regularized partial correlation under different temporal filtering choices. We evaluate features derived from these methods for discriminating states using MVPA. We find that multidimensional parcellations derived from functional data performed similarly, outperforming an anatomical atlas, with correlation and partial correlation (*p* < .05, FDR). Partial correlation, with appropriate regularization, outperformed correlation. Amplitude and covariance generally discriminated less well, although gave good results with high‐dimensionality ICA. We found that discriminative FC properties are frequency specific; higher frequencies performed surprisingly well under certain configurations of atlas choices and dependency measures, with ICA‐based parcellations revealing greater discriminability at high frequencies compared to other parcellations. Methodological choices in FC analyses can have a profound impact on results and can be selected to optimize accuracy, interpretability, and sharing of results. This work contributes to a basis for consistent selection of approaches to estimating and analyzing FC.

## 1 INTRODUCTION

The mapping and analysis of correlated brain activity patterns present in functional magnetic resonance imaging (fMRI) recordings has widespread applications including the investigation of the organization of cognitive processing, the decoding of brain states, and the development of biophysical models and clinical biomarkers (Barkhof, Haller, & Rombouts, 2014; Castellanos, Di Martino, Craddock, Mehta, & Milham, 2013). Core functional connectivity (FC) methodology consists of the calculation of functional dependencies between neurophysiological (functional) measurements of brain activity fluctuations (Biswal, Yetkin, Haughton, & Hyde, 1995; Smith et al., 2011, 2013). One of the major products of FC analyses of FMRI has been the identification of resting state networks (RSNs) (Damoiseaux et al., 2006). RSNs are correlated patterns of brain activity that are consistently found during rest, and reflect the major functionally specialized brain networks related to cognition (Smith et al., 2009). FC studies often aim to explore interactions both within and between these RSNs and their subnetworks, and how these interactions are modulated by cognitive states or other external factors. FC approaches can inform other methods in estimating effective connectivity (EC), the underlying functional and structural relationships producing correlated brain activity (Friston, 1994, Woolrich & Stephan, 2013). The spatial and temporal complexity of fMRI data is such that characterization of FC remains a crucial component to any investigation of EC.

Despite the widespread use of FC‐based methods, there has been limited standardization or optimization of analysis approaches. There is considerable variability in approaches to selecting nodes, preprocessing the time series data, and measuring dependencies between nodes (Varoquaux & Craddock, 2013). The lack of standard approaches makes analysis design decisions arbitrary, and the results of different studies difficult to compare or integrate.

FC analyses typically begin with a selection of a set of nodes from a parcellation of the brain, to form network nodes or seed regions. There are many choices for parcellation strategy, which can trade off neurobiological interpretability and specificity. One option is to use anatomical parcellations or atlases, such as the automated anatomical labeling (AAL) (Tzourio‐Mazoyer et al., 2002) or the Harvard‐Oxford probabilistic atlas (Smith et al., 2004). However, these methods may not be ideal in the sense that observable anatomical boundaries do not necessarily correspond to functional units (Smith et al., 2013). Alternative approaches use information from functional data. In these, nodes are defined from the analysis of blood‐oxygen‐level dependent (BOLD) activations obtained from a localizer or task‐driven fMRI scan (Lashkari et al., 2012; Thirion, Varoquaux, Dohmatob, & Poline, 2014), or by independent component analysis (ICA) of resting fMRI data (Marrelec & Fransson, 2011; Smith et al., 2011). Other, more recent approaches use information from multiple modalities to create parcellations (Glasser et al., 2016). In addition to the nature of defining the parcellations, the level of granularity of the parcellation (i.e., dimensionality) may also have substantial impact on FC analysis results and interpretations. For example, using ICA, it has been observed that in low dimensional parcellations, each component typically represents an extended “entire” brain network, whereas in high‐dimensionality parcellations the obtained components are smaller and they are more likely to represent subparts of networks (Abou Elseoud et al., 2011; Kiviniemi et al., 2009). The choice of the dimensionality will depend on the application or the objective of the study. It has been shown that higher dimensionalities can provide better discrimination between states, particularly if the final analysis is to be a “nodes and edges” network modeling, as opposed to voxel‐wise spatial mapping (Duff, Makin, Madugula, Smith, & Woolrich, 2013).

The frequency dependence of connectivity signals remains relatively poorly explored. FMRI time series activity will combine a variety of signals across a wide range of frequencies, particularly with the development of fast multiband sequences (Feinberg et al., 2010; Moeller et al., 2010). Analyses often apply some band pass filtering in preprocessing to reduce artifacts and target functional activity; however, optimal choices are not well‐established. Although the dominant signal contribution to FC patterns is typically found in frequencies below 0.1 Hz (Cordes et al., 2001), there is a variety of evidence suggesting that fMRI FC is the result of processes occurring at a wider range of frequencies. Meaningful RSN patterns have been observed even after removing all contributions below 0.25 Hz from the fMRI data (Boubela et al., 2013), and some authors have described functional integration of the RSNs at multiple frequency bands up to 0.75 Hz (Gohel & Biswal, 2014) and 1.4 Hz (Kalcher et al., 2014). At higher frequencies, neural signals are more difficult to isolate at least in part due to the slow hemodynamics and higher levels of physiological noise (Cordes et al., 2001; Niazy, Xie, Miller, Beckmann, & Smith, 2011).

Finally, there are numerous choices for the statistical dependency measure used for FC. Correlation—the simplest measure of pairwise similarity between two timeseries–is a generic choice and it is the most widely used measure in FC. However, it is sensitive to global signals and SNR variations, and it cannot be used for distinguishing direct from indirect influences (Cole, Yang, Murray, Repovš, & Anticevic, 2016; Duff, Makin, Smith, & Woolrich, 2018; Friston, 2011). Partial correlation (PC) provides a measure of the statistical dependence between two regions after removing mutual effects from other nodes, reducing some of these issues (Marrelec et al., 2006). PC is commonly estimated via the inverse covariance (i.e., precision) matrix and often requires the use of a regularization technique, as the limited number of time points in the fMRI data sets leads to poorly conditioned inverse covariance matrices. In such cases, a penalty is applied to regularize off‐diagonal elements of the precision matrix (Varoquaux & Craddock, 2013). The choice of the regularization method and the parameters used can affect the structure of FC and its ability to discriminate states (Brier, Mitra, McCarthy, Ances, & Snyder, 2015; Duff et al., 2013; Smith et al., 2011). Besides correlation and PC, other dependency measures have been used. Such measures may involve higher‐order statistics or temporal/phase lag to estimate directionality and temporal causality of connections. These include mutual information (Shannon, 1948) and Granger (lag‐based) causality (Granger, 1969). However, it has been shown that they may perform worse for modeling fMRI connectivity than correlation (Smith et al., 2011). FC changes have been linked to changes in amplitude or power (Duff et al., 2008, 2018; Yang et al., 2007). Cole et al. (2016) recently demonstrated an approach combining covariance and correlation.

The various analytic choices are likely to have interdependent effects on outcomes. Here, we focus on combinations of choices that enable optimal discrimination between brain states. Discrimination is a key outcome for applications that ultimately aim to provide predictions relating to, for example, health outcomes. We assess the specific conditions that optimize discrimination, and their implications for the sources of FC. To date, several studies have separately assessed some of these aspects, such as the performance of partial correlation compared to full correlation (Duff et al., 2013; Smith, Beckmann, et al., 2013), the effects of different preprocessing choices of FC (Shirer, Jiang, Price, Ng, & Greicius, 2015), and differences across region‐definition approaches using activation data (Craddock, James, Holtzheimer, Hu, & Mayberg, 2012; Shirer, Ryali, Rykhlevskaia, Menon, & Greicius, 2012). In a recent study, Abraham et al. (2017) performed a methodological investigation of resting‐state FC pipelines by studying the effects of region extraction, time series estimation, functional interactions, and classification models. They found that the region definition step has the strongest impact on classification, with atlases defined from clustering approaches of resting‐state data, both derived from their own study or defined on large available data sets, as being the best choice among all the options tested. The authors also explored the effect of varying the number of regions in the parcellation, and found an optimal performance with 84 regions.

One reason for the lack of detailed validation of FC analysis methods is that there has been relatively limited data available in which FC has been robustly modulated by basic task conditions where the localization of underlying brain processes are reasonably well understood (Duff et al., 2008; Shirer et al., 2012; Zabelina & Andrews‐Hanna, 2016). FC studies have tended to focus on clinical conditions, where the expected differences in connectivity are unclear and in many cases expected to be subtle. These do not necessarily provide optimal data sets for assessing the sensitivity and interpretability of analysis methods. In this work, we explore the ability of different analysis choices to distinguish fMRI data measuring simple steady state active task conditions involving motor and visual processing, where underlying brain processes are reasonably well understood and localized. The use of active conditions allows controlled manipulations of FC associated with steady state brain activity. The assessment of these steady state conditions provides a simulation of possible differences seen in the resting state in experimental studies, for example across patient groups, or across a learning period. In larger data sets, such as the Human Connectome Project (HCP), fMRI acquisitions consist of pure resting state data–in which classification between states cannot be easily assessed–or block design task‐fMRI data where periodic task switches confound measures of FC. Data with event related trials or block design stimuli, which introduce shifting mean signal levels and transient effects, will grossly affect measures of functional connectivity (Kwon, Watanabe, Fischer, & Bartels, 2016); in other words it becomes hard to disentangle changes in connectivity from changes in coactivation.

The aim of the work is to provide a focused survey of the impact some major analysis choices, and their interactions to complement broader studies that explore the performance of classifiers across larger clinical data sets (Abraham et al., 2017).

## 2 METHODS

### 2.1 Overview

**Figure 1:**
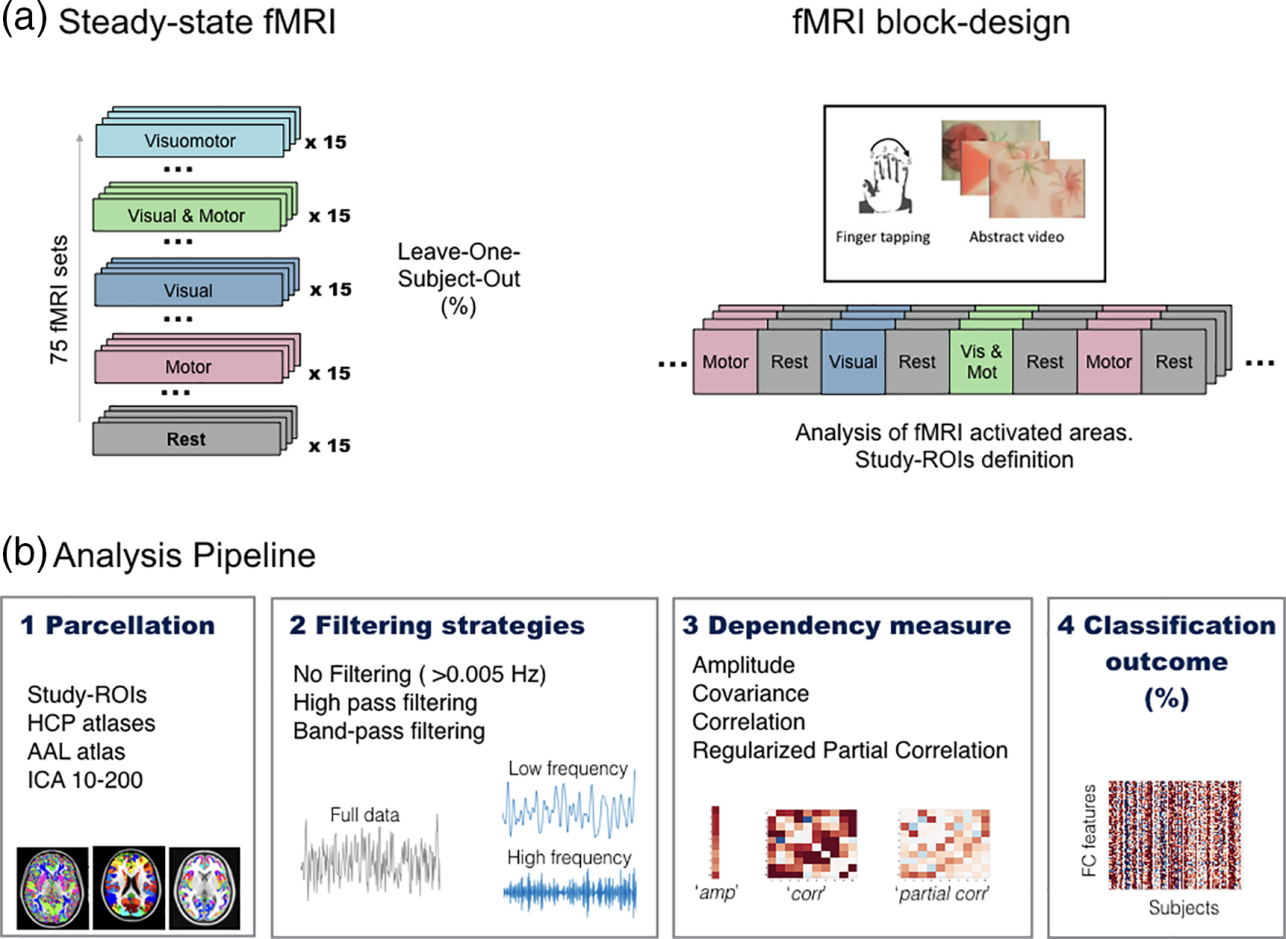
(a) Task design for the fMRI acquisition, and (b) main analysis pipeline including the methodological choices along the process [Color figure can be viewed at http://www.wileyonlinelibrary.com]

The aim of the study was to assess different Functional Connectivity analysis pipeline choices for their ability to produce FC data that would be discriminative of different states. We utilized specifically acquired fMRI data that recorded 15 subjects under five separate steady‐state conditions. These conditions corresponded to the continuous performance of different tasks that elicit activation of well understood networks. FMRI data from a localizer scan was also acquired and used for ROI definition (Figure 1a). The basic pipeline for the analysis is shown in Figure 1b. We separately assessed methodological choices for (1) parcellation, (2) bandpass filtering, and (3) FC dependency measures. We assessed each step separately, utilizing the performance of a multivariate classifier to discriminate between states as a key metric.

### 2.2 Study design and data acquisition

Sixteen healthy volunteers (7 females, 8 males, mean age = 27.25 years, 10 right handed) without previous neurological disorders were initially included in the study after providing written informed consent, in accordance with NHS national research ethics service approval (10/H0707/29). One subject was excluded a posteriori, leaving a total sample of 15 subjects. All of them were scanned under five separate 5 min steady‐state conditions, with no baseline epochs: rest (eyes open), visual only, motor only, simultaneous (but independent) visual and motor tasks; and a combined condition involving a visually cued task, where subjects were instructed to change direction of their tapping when they observed visual features in the video. The visual conditions consisted of videos of colorful abstract shapes in motion designed to be consistent in their visual properties over time. The motor conditions involved uncued continuous sequential finger tapping against the thumb, using the right hand, with periodic changes in direction. An additional task‐activation localizer scan, using pseudo‐randomized 30 s block intervals by baseline periods was performed under the same conditions to enable the identification of brain regions changing in average activation levels during these conditions (Figure 1). This set data was specially designed to assess the ability of imaging methods to discriminate between states and it has already been published elsewhere, in two methodological papers (Costa et al., 2015; Duff et al., 2018).

FMRI data were acquired in a Siemens 3 T scanner, using a 32‐channel coil and a high‐resolution (2 × 2 × 2 mm) fast (TR = 1.3 s) multiband (factor 6) whole‐brain acquisition (Feinberg et al., 2010; Moeller et al., 2010). Each steady‐state scan was approximately 5 min (230 time points). The block‐designed fMRI scan was 10 min. A high‐resolution T1‐weighted 3D magnetization prepared rapid acquisition gradient echo sequence (MPRAGE) was acquired with parameters: TR = 2.0 s; TE = 4.7 ms; flip angle = 8°, 1 mm isotropic resolution.

### 2.3 Data preprocessing

FMRI data were preprocessed using tools from FSL‐FEAT (Smith et al., 2004). Standard preprocessing steps included motion correction, brain extraction, fieldmap unwarping of EPI images using FUGUE (Wilson, Jenkinson, & Jezzard, 2002), and spatial smoothing with a Gaussian kernel of FWHM of 2 mm. Additionally, data was cleaned using FIX (FMRIB's ICA‐based Xnoiseifier) (Griffanti et al., 2014) automated denoising. Registration of EPI data to standard MNI space was performed via high‐resolution T1 images using FSL FLIRT and FNIRT (Andersson, Jenkinson, & Smith, 2007; Jenkinson, Bannister, Brady, & Smith, 2002).

### 2.4 Comparison of connectivity analysis choices

#### 2.4.1 Brain parcellations

We compared the ability of a variety of parcellation approaches to generate discriminative FC matrices. We first evaluated a study‐specific set of ROIs defined from the localizer scans (Study‐ROIs). It might be expected that the measurement of FC changes within networks seen to show activations to given conditions should produce accurate discrimination. The ROIs were derived from a FEAT‐based general linear model (GLM) analysis of data obtained from the block‐design localizer scan using the same task stimuli. For this, we identified ROIs from significant activation clusters generated using an *F*‐test across BOLD signal level responses to all tasks. Note that this procedure assessed changes in mean BOLD signal levels (activation), and not interregional correlations within conditions. We derived 33 ROIs that were classified into: Visual Regions, Motor Regions, and Task Deactivated Regions (details in Supporting Information).

Second, we explored parcellations derived from two anatomical atlases. The Automated Anatomical Labeling (AAL) Atlas (Tzourio‐Mazoyer et al., 2002), which includes 58 ROIs in each hemisphere, and the multimodal surface‐based atlas parcellation provided within the Human Connectome Project (HCP) (Glasser et al., 2016), which includes 180 cortical regions in each hemisphere. The HCP atlas was originally defined in gray‐ordinates. Here, as these analyses focused on volumetric data, we converted it into a volumetric parcellation using the workbench analysis suite (Marcus et al., 2013) projecting the parcellation onto the MNI 2 mm template. We examined the performance of a symmetrized version of the HCP atlas consisting of averaging the time series within the same region across hemispheres, the full atlas containing 360 regions and a combined version including subcortical regions defined from the Harvard‐Oxford subcortical atlas (Smith et al., 2004) (results reported in Supporting Information).

We also assessed parcellations derived using FSL‐MELODIC ICA (Beckmann, DeLuca, Devlin, & Smith, 2005) with dimensionalities ranging from 10 to 200. Group‐ICA decompositions were performed concatenating data from all subjects and all tasks. Of the obtained IC sets, components determined to represent nonneural features such as motion artifacts were removed, giving decompositions of 10, 20, 27, 43, 80, 104, and 130 features.

In addition, we tested three further atlases. First, to enable the assessment of the general performance of surface‐derived parcellations, we assessed an atlas obtained by applying surface ICA on the HCP data, at a range of different dimensionalities similar to the volumetric ICA derived from our own study (Smith, Beckmann, et al., 2013). These surface‐defined parcellations were further projected into volumetric space. We also tested a group functional atlas derived from applying a multilevel bootstrap analysis of stable clusters (BASC) (Bellec, Rosa‐Neto, Lyttelton, Benali, & Evans, 2010) on a subset of data from the 1,000 functional connectome project (Liu, Stufflebeam, Sepulcre, Hedden, & Buckner, 2009), which is publicly available and includes parcellations of different dimensionalities. Finally, we included the functional parcellations from Craddock et al. (2012). These were obtained from clustering algorithms with various similarity metrics, including spatial and temporal correlation and averaged in a group level approach. Further details on these three atlases and their implementation within our study set obtained are provided in Supporting Information.

#### 2.4.2 Extracting time‐series

Study‐specific ROIs, group ICA maps, and atlas‐derived masks were projected onto individual preprocessed fMRI data using spatial regression to obtain subject‐specific time‐series of each component. For ICA‐derived maps, the resulting time‐series were obtained through spatial multiple regressions, the first step of dual regression, using the group ICA spatial maps. For atlas‐based parcellations, timeseries were calculated as the average of all voxels in each parcel.

#### 2.4.3 Connectivity structure with correlation and partial correlation

We derived connectivity matrices from each parcellation using the FSLNets toolbox (Smith, Beckmann, et al., 2013). We first assessed the (full) correlation between time series to study whole‐brain connectivity, given that this is the simplest and most commonly used measure of FC. Functional connectivity (FC) between ROIs or nodes was calculated as the correlation between their time series. Correlation matrices were *Z*‐transformed for statistical purposes. Network matrices of differences between tasks were calculated with paired two‐sided *t*‐tests of connectivity features implemented in MATLAB (MathWorks Inc., Natick, MA, USA).

Results were corrected using False Discovery Rate (FDR) (Benjamini & Hochberg, 1995), with *q* = 0.2 and *q* = 0.05. We find connectivity changes between tasks using an FDR of 0.05 and we also explored changes at a lower rate of 0.2 to visualize the broader pattern of connectivity changes between states.

We also explored the capabilities of partial correlation (PC). As estimation of PC from large covariance matrices can be poorly conditioned, we used group regularization using the L2‐norm (also known as Ridge‐regression or Tikhonov‐based regularization). We assessed several regularization values (from 0 to 5 in steps of 0.1) with the optimal amount of regularization calculated by means of cross validation (CV) using a nested leave‐one‐subject‐out loop (see Supporting Information).

### 2.5 Analysis of covariance and spectral properties

#### 2.5.1 Overall variance (power)

In addition to correlation, we calculated the variance (i.e., reflecting amplitude of time series) and covariance of the ROI‐specific time series as stand‐alone features.

#### 2.5.2 Power spectra and temporal filtering

To investigate the frequency structure of FC, and the effects of data filtering, we obtained the estimated power spectral density of the time series. To obtain robust spectral estimates, we used the Welch's overlapped segment averaging estimator with a maximum eight segments with no more than 50% overlap (Welch, 1967). These are used to calculate a set of periodograms that are combined to obtain the final PSD estimate.

Band pass filtering is a common preprocessing step in FC FMRI analyses. A number of temporal filtering strategies were applied to the individual time‐series. We evaluated no filtering (beyond a removal of very low‐frequency drift <0.005 Hz), and we tested high‐pass filtered data at different frequency cutoffs covering the full available range. In addition, we evaluated band‐pass filtered data in four nonoverlapping bands: [0.005–0.096] Hz; [0.096–0.182] Hz; [0.182–0.298] Hz, and [0.298–0.385] Hz. These bands do not cover equal spectral power, but are intended to correspond to frequency bands often explored in practice. For filtering the timeseries, we used Butterworth filters of order N = 4 implemented in MATLAB.

### 2.6 Classification

We assessed the discriminative capabilities of connectivity features using a multiclass linear Support Vector Machine (SVM) classifier (Chang & Lin, 2011) with a leave‐one‐subject‐out (LOSO) cross‐validation approach, both implemented in MATLAB. At each iteration, the 5 acquisitions of a given subject were separated to be used as a test set and the remaining subjects were used for training in order to estimate the SVM model. For SVM, we evaluated eight soft margin C‐values between 0.001–1,000 to ensure maximal discrimination for each data input. Overall, predictions were consistently maximized for values of soft margin parameter C > =1. When PC regularization was used, we selected the best regularization with cross‐validation within the training set. For each assessment of classification performance, all features from connectivity (correlation and partial correlation) or power/variance were included for classification. Before model estimation, features from the training set were normalized within subjects. We obtained normalized features as the ratio between each subject and task specific connectivity network and the average connectivity network of the same subject across the five tasks. While it was not the core focus of this work, we also tested whether k‐NN and random forest (RF) classifiers would produce similar results (Mitchell, 1997).

### 2.7 Summary scores and differences between methods

Final classification performance scores were computed as the percentage of correct classification across tasks and subjects. The total number of samples was 75 to be classified into five tasks. Chance performance was therefore at 20%. That is, one would expect a classification rate of 1/5 by chance. We used McNemar's test to obtain statistics for the comparison of error rates between the different assessments of classification (Fleiss, 1981). This test is used to evaluate the improvement in correct classification between two methods based on their error rates given the actual observed categories.

We sequentially performed a series of assessments: (1) across parcellations using correlation and minimal filtering, (2) between correlation and partial correlation for each given parcellation, (3) across parcellations using partial correlation, and (4) between methods and parcellations with filtered data.

Given the many possible permutations of analysis settings, and expected random variation in performance of individual pipelines, we did not aim to identify a single optimal pipeline, or make definitive comparisons between individual permutations of pipelines. Instead, we assess separate pipeline elements sequentially, identifying general patterns of effects. To control false positives, we use False Discovery Rate (FDR) with *q* = 0.05, and test for robustness to variations of parcellation schemes using the AAL as a baseline (i.e., which parcellations give significantly higher predictions than AAL?), while keeping the other pipeline components fixed.

When assessing overall differences between correlation and partial correlation across parcellations (i.e., does partial correlation proves a better prediction?) we used a pairwise Wilcoxon signed‐rank test.

### 2.8 Combining pipelines

Features from different analysis pipelines could be complementary—for example, large and fine scale parcellations, or features in different frequency bands. We therefore assessed the value for discrimination of conditions using concatenations of features generated by different analysis pipelines.

## 3 RESULTS

### 3.1 Standard correlation based analysis of test data

**Figure 2:**
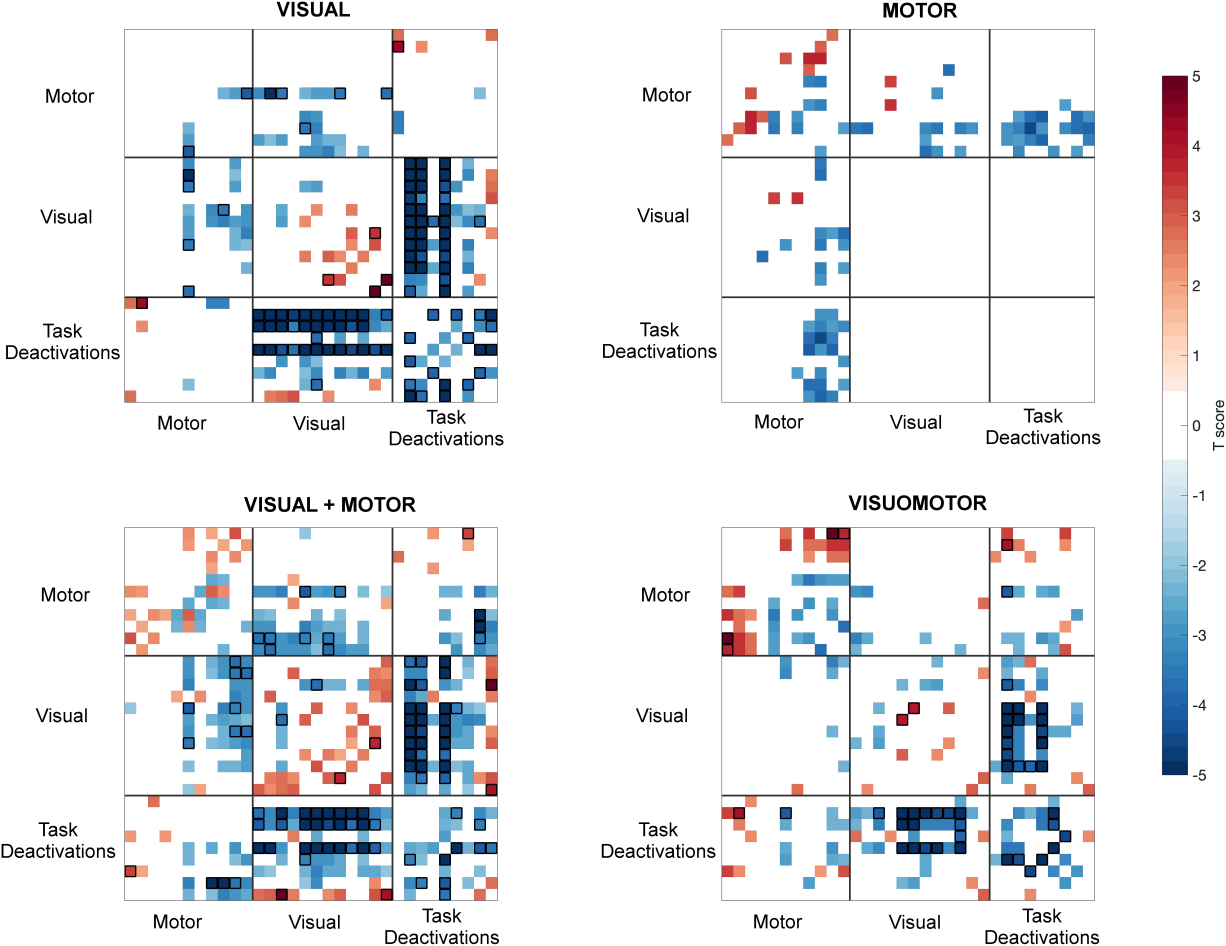
Overall pattern of connectivity changes between the active tasks and rest using the study‐ROIs. All connectivity maps are thresholded using FDR with *q* = 0.05 (highlighted with unfilled black squares). Connectivity differences with *q* = 0.2 are shown on the background using blue‐red scale for decreases (blue) and increases (red) with respect to rest [Color figure can be viewed at http://www.wileyonlinelibrary.com]

Standard correlation‐based FC assessments identified distinct differences in functional connectivity across the resting, visual, motor and combined states. With respect to rest, the visual condition produced increases in correlation within the visual cortex, and decreases in correlation of these regions with other brain regions, including parts of the motor network and regions associated with task‐related deactivations. The motor task did not show significant changes compared to rest at an FDR q value of 0.05. However, at a FDR q of 0.2, a pattern of reduction of FC between regions of the motor network with the visual and task deactivated regions, and increases in some motor connections, including the connections from cerebellum and putamen with the motor cortex. The two tasks combining visual and motor conditions produced an extensive pattern of connectivity changes with respect to rest, which broadly reflected a combination of effects of the visual and motor conditions, together with additional differences in the connections showing interactions between systems (Figure 2 showing FDR‐threshold differences using parcellations from Study‐ROIs at *q* = 0.05 with the pattern at *q* = 0.2 at the background). To assess the effects of head motion on discrimination, we used individual summary measures of motion as classification features in the same SVM algorithm. These were not predictive of brain states (accuracy 20% chance), indicating that our results are not likely to be driven by differences in head motion during the scanning session.

### 3.2 Assessment of Parcellations using correlation

**Figure 3:**
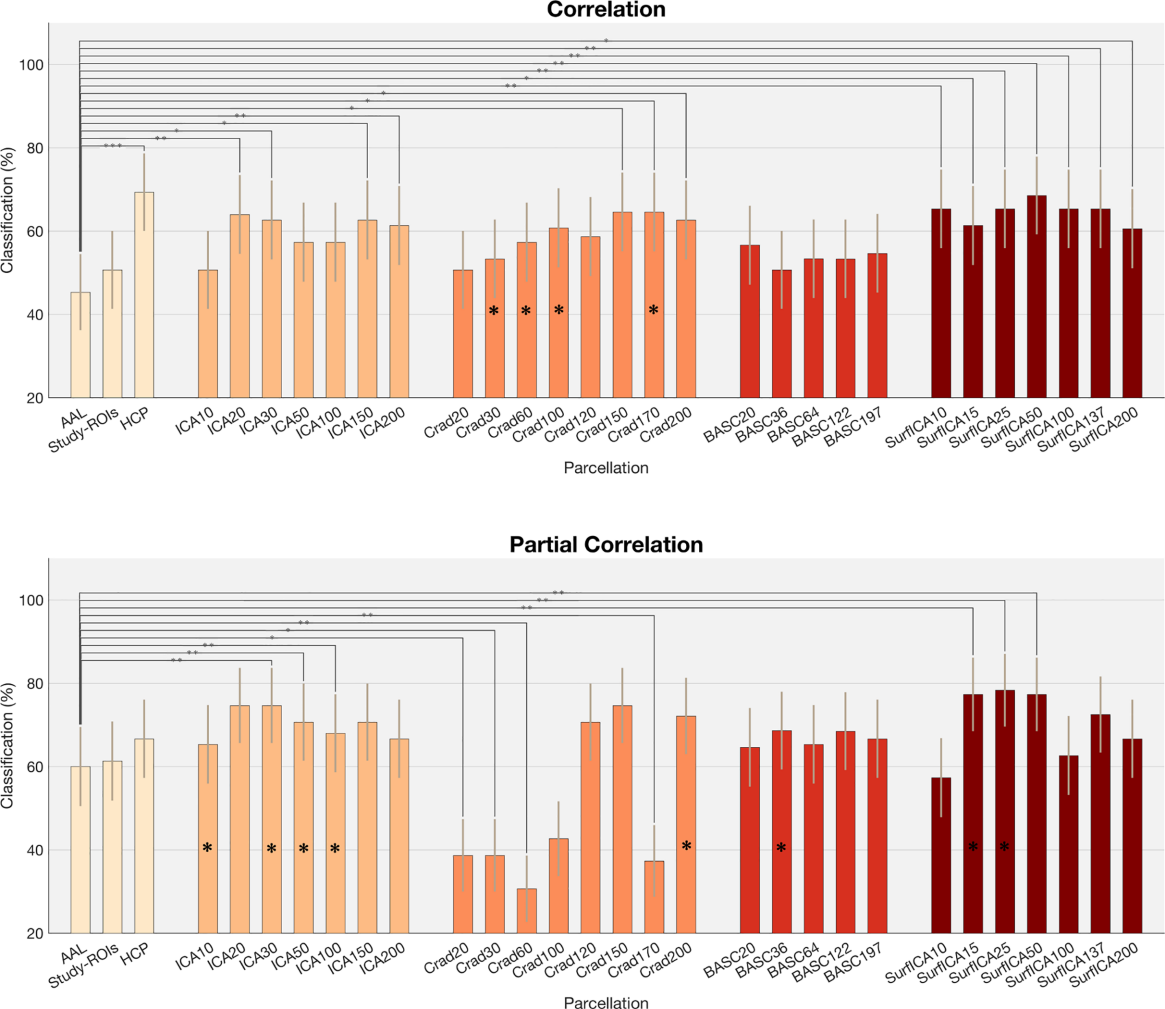
Classification rates for all the parcellations tested. (a) Using correlation as the dependency measure and (b) using regularized partial correlation as the dependency measure. For comparisons across parcellations, * indicates *p* < .05, ** indicates *p* < .01, and *** indicates *p* < .001. For comparisons between methods, * inside the bars indicates significant differences between full correlation and partial correlation at *p* < .05 level. Results are reported here as uncorrected *p* values. Refer to main text for comparisons surviving FDR threshold [Color figure can be viewed at http://www.wileyonlinelibrary.com]

We evaluated the effect of parcellation on between state discrimination using correlation as a dependency measure. We compared the performance of the different parcellation approaches using the AAL atlas as the baseline parcellation. Results are summarized in Figure 3a. The HCP parcellations performed significantly better than the AAL atlas (*p* = .00045). Using ICA, we obtained an accuracy of 51% for ICA10 and values around 60% for the rest of ICA dimensionalities. ICA parcellations of dimensionalities 20, 30, 150, and 200 performed significantly better than the AAL atlas (*p* = .008, *p* = .020, *p* = .0175, and *p* = .008). All results were significant for an FDR of *q* = 0.05.

The two multiscale parcellations derived from functional data—the Craddock and BASC parcellations–also performed similarly (accuracies around 60%). In this case, the Craddock atlas of dimensionalities 150, 170, and 200 performed significantly better than AAL (*p* = .01, *p* = .01, and *p* = .0102).

Surface‐ICA of all dimensionalities performed better than AAL parcellations (*p* = .008 for surface‐ICA10, *p* = .02 for surface‐ICA15, *p* = .0053 for surface‐ICA25, *p* = .0022 for surface‐ICA50, *p* = .0067 for surface‐ICA100, *p* = .004 for surface‐ICA137, and *p* = .017 for surface‐ICA200). All significant for an FDR of *q* = 0.05.

### 3.3 Partial correlation and regularization techniques

We tested partial correlation for its discriminative capabilities, and explored the role of regularization on this outcome. Regularization imposes sparsity on the obtained partial correlation matrices, avoiding overfitting. As different levels may be optimal for different parcellations, we evaluated performance with various regularization levels and we selected the optimal regularization for each parcellation by using a Leave‐one‐subject‐out (LOSO) cross‐validation approach. We report the mean, standard deviation and the range of the optimal regularization scores for HCP, AAL, Study‐ROIs, and ICA parcellations in Table 1. In general, atlas‐based parcellations and Study‐ROIs required higher regularization than ICA parcellations of similar number of features, perhaps indicating these parcellations are of lower rank. For the HCP atlas, we obtained optimal regularization using high regularization scores. We observed that across ICA dimensionalities, the level of regularization needed was higher for higher number of components (See detailed results in Supporting Information).

**Table 1:** Summary of optimal regularization levels across parcellations

	Mean	Range (min‐max)
*HCP*	7.36	5.5–8
*AAL*	2.37	1–5
*Study‐ROIs*	1.90	0.5–4.9
*ICA10*	0.61	0.3–1.1
*ICA20*	0.95	0.1–2.3
*ICA30*	2.94	0.4–4.3
*ICA50*	0.88	0.3–1.8
*ICA100*	1.39	0.6–4.4
*ICA150*	1.48	0.3–2.4
*ICA200*	2.23	0.3–3.9

### 3.4 Assessment of Parcellations using partial correlation

The results of a pairwise Wilcoxon signed‐rank test indicated that across parcellations, partial correlation tended to improve prediction accuracy compared to full correlation (*p* = .045). The best performances were obtained with ICA20 and ICA30 parcellations (74.67%). Compared with the AAL, we observed statistically higher performance for ICA30, ICA50, ICA100, (*p* = .008, *p* = .003, *p* = .0058). Surface‐ICA of dimensionalities 15, 25, and 50 also outperformed AAL (*p* = .004, *p* = .001, *p* = .004). Craddock parcellations of dimensionalities 20, 30, 60, and 170 performed worse than AAL. All results were significant for an FDR of *q* = 0.05.

### 3.5 Assessment of Parcellations using amplitude and covariance

To some extent, discrimination obtained with correlation or covariance measures could be produced by simple changes in the amplitude (i.e., variance) of local signals, rather than intrinsic changes in connectivity. To assess this, we assessed the extent to which amplitude and covariance features from the different parcellations performed as features for classification.

In general amplitude performed poorly for the majority of the parcellations, except for ICAs of higher dimensionalities (>100 components), where performance was around 60%, similar to the values obtained in correlation. ICA of dimensionalities 150 and 200 performed significantly better than AAL (*p* = 6.5 × 10^−6^ and 2.7 × 10^−6^, surviving an FDR threshold of *q* = 0.05).

**Figure 4:**
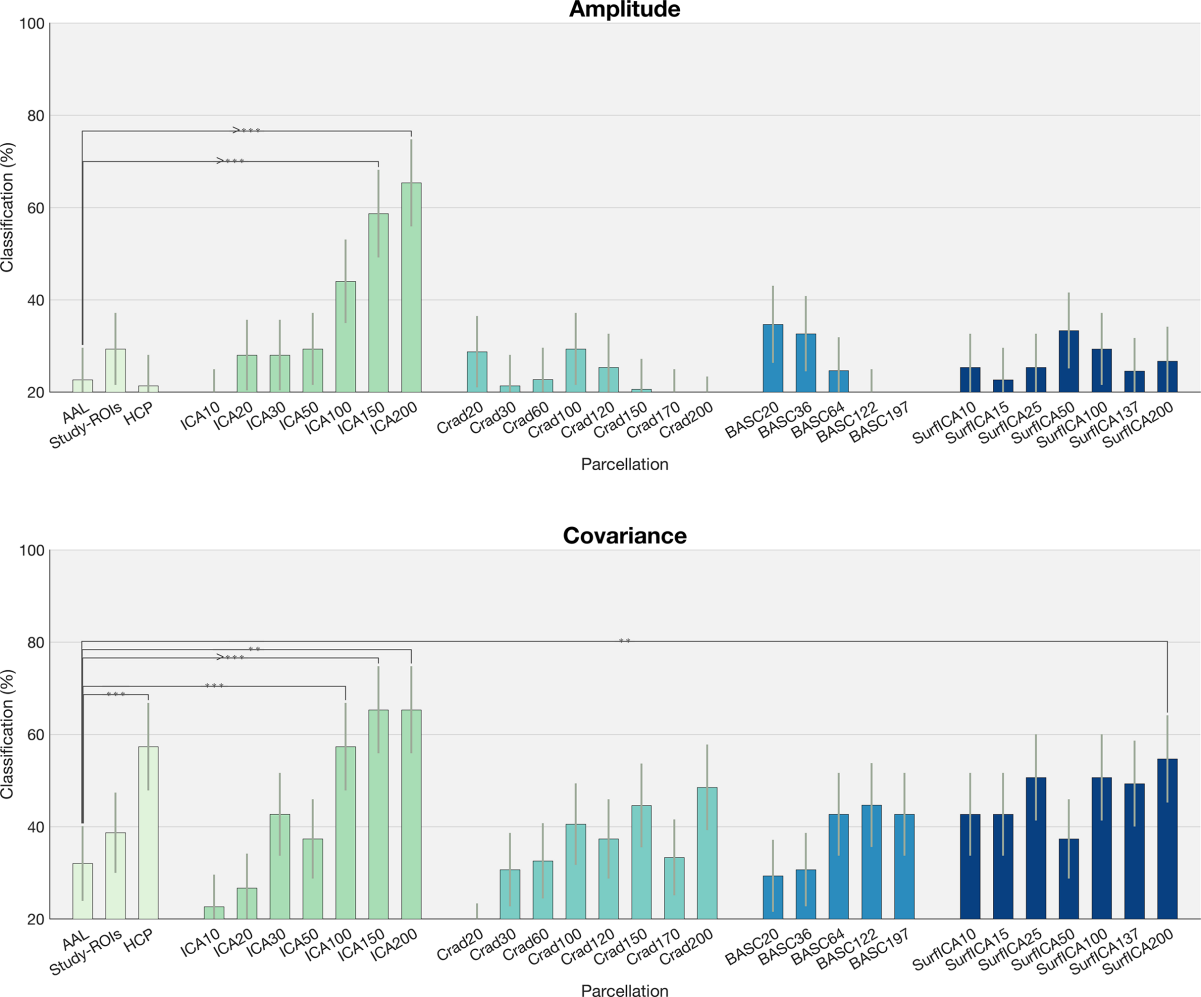
Classification rates for all the parcellations tested. (a) With amplitude as the dependency measure and (b) with covariance as the dependency measure. For comparisons across parcellations, * indicates *p* < .05, ** indicates *p* < .01, and *** indicates *p* < .001. Results are reported here as uncorrected *p* values. Refer to main text for comparisons surviving FDR threshold [Color figure can be viewed at http://www.wileyonlinelibrary.com]

For covariance, we observed in general poor performance for many of the parcellations but significantly higher performance, when compared with the AAL atlas, for the HCP parcellation (*p* = 9.4 × 10^−4^), ICA of dimensionalities 100, 150, and 200 (p = 6.5 × 10^−4^, *p* = 2.1 × 10^−4^, and *p* = 2.1 × 10^−4^,) and surface‐ICA of 200 features (Figure 4). All significant for an FDR of *q* = 0.05.

### 3.6 Assessing frequency specificity and filtering choices

Functional connectivity measures will be affected by a variety of processes producing BOLD signal variation across a range of frequencies. Temporal filtering is often used prior to FC estimation to isolate informative signals. We explored the frequency specificity of connectivity signals, and their effects on discrimination. A variety of high‐pass and band‐pass filter settings are used in FC analyses. We assessed the effects of varying the high‐pass filter cut‐off for the BOLD time‐series.

**Figure 5:**
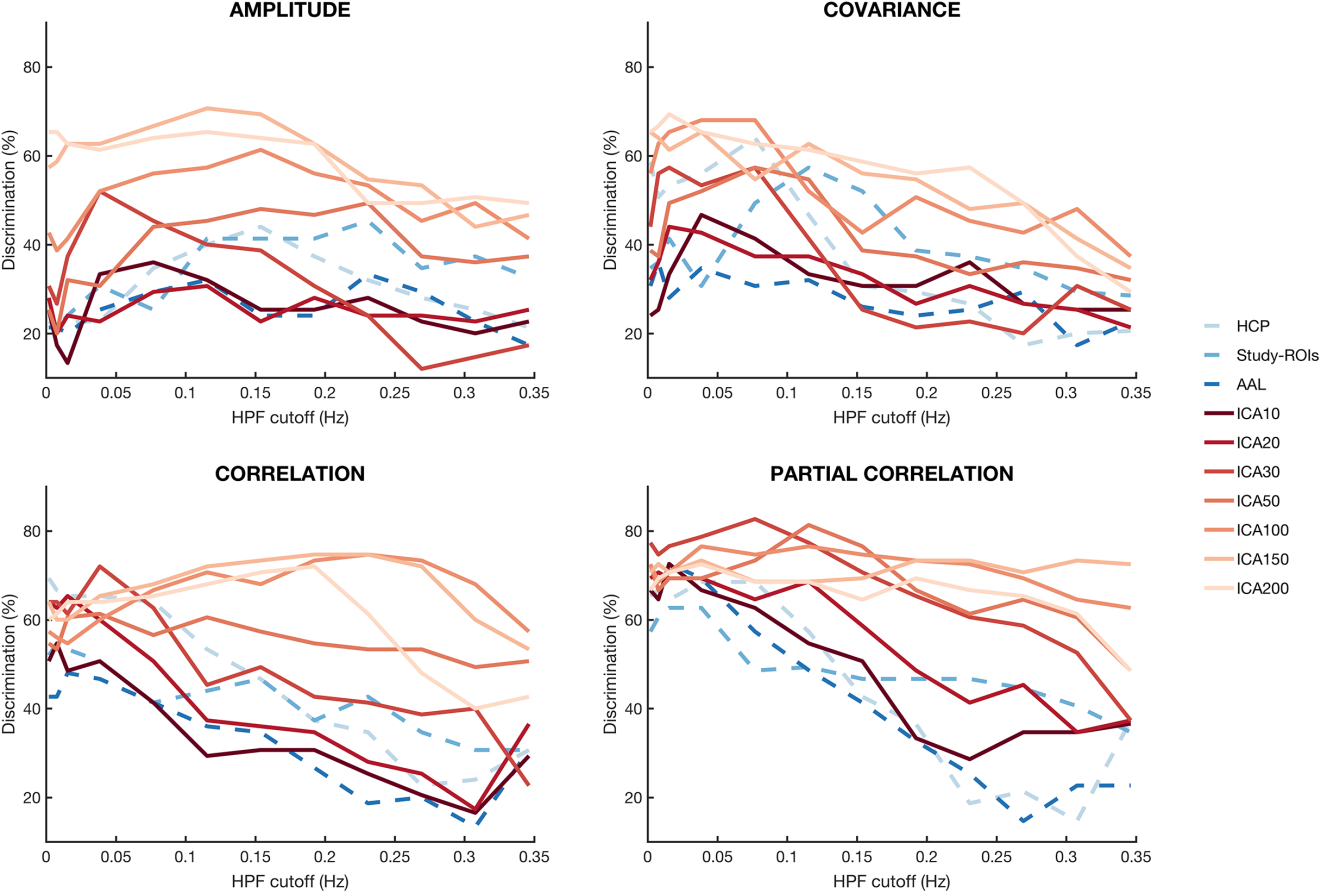
Classification rates obtained using different HPF cutoffs for amplitude, covariance, correlation, and partial correlation measures. For each dependency measure, we evaluated classification using filtered data across a set of HPF cutoffs, from 0.005 to 0.375 Hz. The different parcellation schemes are coded in red for ICAs and in dashed blue for the anatomical and functional atlases [Color figure can be viewed at http://www.wileyonlinelibrary.com]

With correlation and partial correlation, we observed a drop‐off in performance at higher HPF cutoffs for low‐dimensionality ICA and atlas‐based parcellations. However, for ICA parcellations of high dimensionality, classification performance remained high even with very high frequency cutoffs. Interestingly, this effect was observed in partial correlation but not in correlation, where a drop‐off in discrimination was observed at HPF levels around 0.25 Hz. Similar patterns were observed for amplitude and covariance, but were not observed in other non‐ICA parcellations (Figure 5).

We also assessed the effects of band‐pass filtering on discrimination, using four distinct bands. Classification results at the lowest frequency bands were similar to those obtained with nonfiltered data for most parcellations. However, similar to the results obtained with high‐pass filtering, we observed that higher frequency bands were generally less discriminative. However, as for the high‐pass filter, high‐dimensionality ICA parcellations remained reliably predictive even when only high frequency data was available (Supporting Information).

The discrimination results concurred with changes in the spectral profiles of nodes across tasks (Supporting Information). All regions showed the greatest changes in the lowest frequencies (i.e., <0.1 Hz), where power was greatest. Changes in this frequency band tended to be in concordance with the overall time series—significant increases and decreases during visual and motor tasks, respectively, compared to rest (Supporting Information).

### 3.7 Concatenation of features

We tested whether different choices of parcellation and frequency band may contain complementary information to differentiate brain states. For this, we created sets of features combining different atlases, dimensionality, and temporal filtering. Overall, combining features did not produce significant performance boosts over the best single‐choice features (data not shown).

### 3.8 Choice of classifier

We also performed our classifications using K‐NN and random forest classifiers instead of SVM. Results followed the same pattern, although overall scores were consistently lower than SVM (Supporting Information).

## 4 DISCUSSION

This work contributes to the comparison and identification of optimal strategies for functional connectivity analysis (Abraham et al., 2017; Shirer et al., 2012). We aimed to perform a detailed assessment of key choices in functional connectivity analysis: brain nodes, dependency measures, and temporal filtering and the frequency specificity of signals. In contrast to studies of such choices on data from clinical research, we focused on analyzing simple, well‐controlled task‐conditions with predictable loci of activity and reliable changes in connectivity. We assessed these choices for their impact on discriminability and interpretability, and explored how these different choices interacted in their outcomes. Overall, we found important differences in the discriminability of different analysis choices, and obtained results providing insight into how these choices combine.

Assessing analysis pipelines must take into account the risk of over‐interpreting results that may be driven by noise and multiple assessments. We utilized FDR to limit false positives, and focus on reporting overall patterns of the effects of different pipeline choices, rather than making strong claims regarding optimal choices.

We found that many functionally derived parcellations outperformed the AAL atlas, both with correlation and partial correlation. These included HCP‐defined parcellations, ICA of different dimensionalities and other functionally defined multidimensional atlases. ICA parcellations, but not the HCP‐derived parcellations, show some benefit from the use of partial correlation and temporal filtering. ICA parcellations provided signals with far greater predictive information in higher frequency bands, which could be important in specific applications.

Parcellation choice had a major impact on discriminability. ICA provided strong discrimination, particularly at higher dimensionalities, and was the best choice when partial correlation was used as dependency measure. Lower dimensional ICA parcellations typically include the well‐studied resting‐state‐networks (RSNs), which are split at higher dimensions (Kiviniemi et al., 2009; Smith et al., 2009). ICA can be expected to identify distinct signal components, without redundancy across nodes. However, the interpretability of measured connectivity between these networks is complicated by the extended nature of the networks, and possible dependences on specific constraints imposed in their generation (Abraham et al., 2017; Gonzalez‐Castillo et al., 2015).

The HCP multimodal parcellation, defined by an extensive bespoke integration of a variety of modalities including high‐quality functional imaging of resting state and task activation and measures of structural architecture (myelin and cortical thickness), also provided good results, producing high discrimination accuracies, and outperforming AAL and Study‐ROIs, with full correlation. The HCP atlas is defined on the cortical surface (Glasser et al., 2016), while our data are defined in the 3D volume space. To overcome this, here we projected the surface template parcellation onto a volume, which is likely to be nonoptimal. Preprojecting functional data onto the surface could further improve the performance of the HCP atlas. Partial correlation did not improve the results of the HCP atlas, possibly due to a higher level of redundancy of signals across the many regions making partial correlation estimates less stable (i.e., driven by noise). The HCP atlas also appeared to be more sensitive to the choice of filter cutoff, indicating that some of the regions might be heterogeneously affected by high frequency noise. In summary, HCP parcellation outperformed the AAL atlas and Study‐ROIs and performed similarly to the best performing ICA parcellations, although may be more sensitive to temporal filtering. However, its state‐of‐the‐art biological validity and its broad general applicability compared with ICA maps suggest that it might be recommended as an interpretable and high‐performing parcellation.

We also used a simple data‐driven parcellation derived from task activation and deactivation maps derived in a separate scan (Study‐ROIs). Despite being directly derived from task‐related activity, it provided weaker discrimination. This suggests that connectivity changes may be more extensive or complex than changes in task activation, and warns against driving connectivity analyses node selection from task‐activation results. The HCP atlas also includes information from task‐activation data. However, in HCP, the parcellation is defined based on input a broad range of modalities, including resting‐state fMRI and structural information, which may enhance its discriminative capabilities.

We observed covariance and even node power (amplitude) alone performing well with ICA parcellations of high dimensionality. This supports recent reports of tight theoretical and empirical associations between signal variance amplitudes and functional connectivity in FMRI (Bijsterbosch et al., 2017; Cole et al., 2016; Duff et al., 2008, 2018).

We found partial correlation to perform better than full correlation in many of the parcellations tested, with overall significantly better performance. This is in agreement with previous results obtained from simulations (Smith et al., 2011; Wang, Kang, Kemmer, & Guo, 2016) and it adds evidence to the advantages of partial correlation summarized elsewhere (Varoquaux & Craddock, 2013)., The benefits that we observed were largely only present for ICA parcellations. In the spatial ICA used for FMRI data, components are defined from a rotation of an initial principle component data reduction that maximizes explained variance by the N components (Beckmann et al., 2005). Thus, every IC must explain substantial unique signal variation. Anatomically defined atlases may define nodes with very similar functional properties. In particular, they often define separate nodes in the left and right hemispheres that in many states contain almost identical functional signals. Redundancies across nodes may make the estimation of partial correlation matrices challenging. These empirical results suggest promise that underlying network dependencies can be estimated from resting state data.

We found evidence that FC varies across different frequency bands. As the largest amount of power in resting‐state fMRI is typically concentrated in lower frequencies (<0.1 Hz), connectivity analysis has typically focused on these low frequencies (Cordes et al., 2001). In our study, we found that higher frequencies performed surprisingly well under certain configurations of atlas choices and dependency measures. Similar to Cordes et al. (2001), we find high performances when low frequencies are included and a gradual decline in performance at higher HPF cutoffs. However, the use of partial correlation maintains performance at high levels even at high cutoffs. This pattern is only observed when high dimensionality ICA parcellations are used. ICA of low dimensionalities and all atlas parcellations—including HCP, showed worse discrimination with higher HPF cutoff. This result suggests an additional ability of high‐dimensionality ICA to isolate information from noise—typically found in this range of higher frequencies (Griffanti et al., 2017). These results support previous findings from the FC literature that point to a rich distribution of FC information across frequencies. Richiardi, Eryilmaz, Schwartz, Vuilleumier, and Van De Ville (2011) used wavelet decomposition and found that even if the main discriminative connections were reported in the lowest frequencies, the combination of information from different bands improved classification results. Kwon et al. (2016) studied attention and visual tasks using long‐trial designs and found that connections between visual and default mode networks occurred at low‐frequencies, whereas connectivity of subregions within the visual system was driven by high frequencies, they suggested a dichotomy between long‐range connectivity across larger networks and short‐range connectivity within the sensory network. Another study, using fMRI in combination with EEG, has suggested the applicability of fMRI in detecting higher frequency information to map neural oscillations throughout the brain (Lewis, Setsompop, Rosen, & Polimeni, 2016). In our study, we found an interaction between ICA dimensionality and frequency; higher ICA dimensionalities showed high discriminability at high frequencies. We speculate that partial correlation matrices obtained from these higher ICA parcellations may contain information about fine interactions within subnetworks of larger brain systems, similar to what were reported by Kwon and colleagues. While high dimensional ICA parcellations provided high discrimination even at high frequencies, all other parcellations had substantially poorer discrimination in this band. This suggests that ICA may successfully spatially separate complex patterns of signal, retaining high frequency information that is averaged out with other parcellations. However, care is required to ensure that these are likely to be neural in origin.

In addition, the observed higher discrimination could be due to a rank effect, given that connectivity matrices obtained from ICA features are likely to be better‐conditioned than the other parcellations tested.

We tested the performance of concatenated covariance and correlation matrices and we did not observe significant increases in performance. Although one would predict that concatenation of matrices could improve performance given that correlation and covariance carry different information about brain states, we believe that we were not able to detect such effect possibly because of a ceiling effect in our classifier and the relatively small number of subjects tested.

Our study has some limitations. First, further studies should increase the number of subjects and conditions, with more advanced assessment methods, to explore combining different data. Another caveat is that we reached a ceiling of discrimination, in the sense that many of the combinations that we tried maximized prediction accuracies, without leaving enough space for observing an improvement in classification. There are also some methodology‐related limitations, for example all parcellations were tested on the fMRI volumetric space, even when the HCP atlas parcellation and the HCP‐derived ICA maps were originally defined in the cortical surface space. We believe that future work should focus on extending FC methods to map directly the parcellations to the surface.

Our active‐state conditions provide reliable, localized modulations of steady‐state FC that are ideal to assess methods. The changes in FC induced by these tasks affect major RSNs in a similar way to putative changes produced by disease or other factors, but are more robust and localized (Fransson, 2006; Harrison et al., 2008; Duff et al., 2018). Nevertheless, it is interesting to note that the changes in FC induced by basic tasks remain poorly understood. Assessments of FC using carefully controlled modulations complement assessments using clinical and other experimental data sets where modulations are smaller, less reliable and more prone to artifacts.

Our results complement those previously described in clinical data sets (Abraham et al., 2017), especially those regarding the parcellation choice. Both works agree in recommending data‐driven parcellations based on functional connectivity—both using study data or independent data, rather than other choices based on predefined atlases. The parcellation provided by Craddock performed well in both studies. However, the high classification performance that we observed with ICA was not found in their study. These differences could be due to higher heterogeneity in their data set but also caused by methodological differences. For ICA, they used a fixed number of components selected with nonparametric noise modeling, while we explored a wide range of dimensionalities. Similarly, in the implementation of partial correlation, we provide a deeper investigation using a range of different regularization values.

In conclusion, we have provided empirical evidence showing how different combinations of methodological choices affect the discriminability and interpretability of FC studies, and have provided recommendations for choices of analysis pipelines. FC signals result from interactions of contributions across different spatial, temporal, and frequency ranges. In particular, we found predictive information at higher frequencies under parcellations derived from high dimensionality ICA, corroborating many recent observations (Lewis et al., 2016; Trapp, Vakamudi, & Posse, 2018). Ongoing systematic assessment of FC analysis pipelines on a broad range of data sets will provide an increasingly solid basis for the design and interpretation of these analyses.

## Supporting information

Appendix S1: Supporting Information.
