# Supplementary material for: Spatial parcellations, spectral filtering, and connectivity measures in fMRI: Optimizing for discrimination
Source: Hum Brain Mapp. 2018 Sep 26;40(2):407–19. doi: 10.1002/hbm.24381 (PMC6492132; doi:10.1002/hbm.24381)
Supplement: Supplementary file 1 — Appendix S1: Supporting Information. [file HBM-40-407-s001.pdf]

SUPPLEMENTARY MATERIAL
Spatial parcellations, spectral filtering and connectivity measures in fMRI: optimizing for discrimination.

Roser Sala-Llonch, Stephen M. Smith, Mark Woolrich, Eugene P. Duff

SUPPLEMENTARY METHODS

Detailed information about all parcellation schemes and their variations

Overall, we tested 4 different atlases as well as parcellations derived from task-related activation maps, volumetric-ICA and Surface-ICA algorithms. The main manuscript focuses on a subset of these methods. However, we provide a full description below.

ICA-based parcellations

We used steady-state data, concatenated across tasks and subjects to obtain different sets of independent components. We created ICA decompositions of 10, 20, 30, 50, 100, 150 and 200 ICs. From each of these sets, we excluded components of non-interest, resulting in parcellations of 10, 20, 27, 43, 80, 104 and 130 components.

Study-specific ROIs

We used fMRI data from the block-designed fMRI to generate a set of 33 ROIs. Each region was defined as a combination of group task-activations obtained by GLM-FEAT analysis and atlas-based regions. Regions are listed in Supplementary Table I.

The AAL atlas

This atlas has been widely used in the functional connectivity literature. It is fully described elsewhere [Tzourio-Mazoyer et al., 2002].

HCP parcellation and variations

The HCP atlas is a multi-modal parcellation recently developed within the HCP (Glasser et al., 2016). Variations on the HCP atlas included the symmetrized vs the non-symmetrized version as well as the inclusion of subcortical segmentations. For the symmetrized version, we averaged the equivalent timeseries across hemispheres, resulting in 180 nodes. For the full version we used each region independently, giving 360 nodes.

Surface-ICA

We tested surface-ICA maps derived using the HCP procedures [Smith et al., 2013a]. These were obtained by running ICA on the CIFTI surface maps. We tested sets of maps with dimensionalities: 10, 15, 20, 25, 50, 100, 137 and 200 features.

Additional atlases.

We tested parcellations obtained from the BASC atlas [Bellec et al., 2010] of dimensionalities 20, 36, 64, 122 and 197. In addition, we tested the parcellation scheme provided by Craddock et al., [2011] of dimensionalities 20, 30, 60, 100, 120, 150, 170 and 200.

ID	Description	Name	RSN
1	Cingulum	Cing	*Task Deactivations*
2	Cuneus	Cuneus	*Task Deactivations*
3	Intracalcarine	IntraCalc	*Task Deactivations*
4	Lateral Occipital Superior Dorsal	LatOccSupD	*Task Deactivations*
5	Lingual Dorsal	LingualD	*Task Deactivations*
6	Occipital Pole Dorsal	OccPoleD	*Task Deactivations*
7	Precuneus Dorsal	PreCunD	*Task Deactivations*
8	Temporal Occipital	TempOcc	*Task Deactivations*
9	Left cerebellum	CbL	*Motor Regions*
10	Right cerebellum	CbR	*Motor Regions*
11	Left putamen	PutL	*Motor Regions*
12	Right putamen	PutR	*Motor Regions*
13	Left Inferior Prefrontal Gyrus	InfPreGL	*Motor Regions*
14	Supplementary Motor Area	SMA	*Motor Regions*
15	Supplementary Motor Area 2	SMA2	*Motor Regions*
16	Right Inferior Prefrontal Gyrus	InfPreGR	*Motor Regions*
17	Left Posterior Gyrus	PostGL2	*Motor Regions*
18	Right Posterior Gyrus	PostGR2	*Motor Regions*
19	Left Premotor Cortex Hand area	PreHL	*Motor Regions*
20	Right Premotor Cortex Hand area	PreHR	*Motor Regions*
21	Left Lateral Occipital	LatOccL	*Visual Regions*
22	Right Lateral Occipital	LatOccR	*Visual Regions*
23	Left Lateral Occipital Superior	LatOccSL	*Visual Regions*
24	Right Lateral Occipital Superior	LatOccSR	*Visual Regions*
25	Left Lingual	LingActL	*Visual Regions*
26	Right Lingual	LingActR	*Visual Regions*
27	Right Occipital Fusiform	OccFusAR	*Visual Regions*
28	Left Occipital Fusiform	OccFusAL	*Visual Regions*
29	Left Occipital Pole	OccPoleL	*Visual Regions*
30	Right Occipital Pole	OccPoleR	*Visual Regions*
31	Left Temporal Occipital	TempOccL	*Visual Regions*
32	Right Temporal Occipital	TempOccR	*Visual Regions*
33	Lateral Inferior Occipital	LatOccInfD	*Visual Regions*

Supplementary Table I: Regions defined within the study-specific ROIs

SUPPLEMENTARY RESULTS

Testing different versions derived from HCP atlas.

We compared the classification performance obtained different versions derived from the HCP atlas, a symmetrized version, one using all the features and one in combination with subcortical segmentations.

We report classification results obtained with amplitude, covariance, correlation and partial correlation for the three versions of the HCP atlas.

We observe that for covariance, correlation and partial correlation, the symmetrized HCP version performed better than the full HCP atlas. For Amplitude, both results were close to chance level, but the full HCP performed better (Supplementary Table II). Adding subcortical structures to the atlas did not improve the results for covariance, correlation or partial correlation. However, the performance for amplitude was significantly higher. We only included the symmetrized version in the manuscript, due to its overall good performance and because it represented less computational load compared to the full version.

	**Symmetrized HCP**	**Full HCP**	**HCP + Subcort**
Amplitude	21.33 %	26.67 %	45.33 %
Covariance	57.33 %	53.33 %	30.67 %
Correlation	69.33 %	65.33 %	37.33 %
Partial Correlation	66.67%	56.50 %	26.67 %

Supplementary Table II: Comparison between symmetrized HCP atlas and two other versions derived from HCP: one using all features from both hemispheres*, and one combined with subcortical parcellations.*

Optimizing regularization levels for each parcellation scheme.

We tested a range of regularization levels and observed good performance at many of them for the different atlases or parcellation strategies (Supplementary Figure 4).

We selected the optimal level of regularization for each parcellation using cross-validation. That is, we selected, across a range of regularization levels, the one being more discriminant in the corresponding training set.

As reported in the main text, the atlases required higher parcellations than the ICA parcellation. Across ICA parcellations, higher dimensionalities needed in general higher regularization. In almost all parcellations the classification results reached the maximum levels at regularization lower than 5. For the HCP atlas, we needed to test a longer range of values, and optimal rhos were found between 7 and 8. In this case, we repeated the full pipeline including cross-validated classification and the results were not significantly higher

(classification=70.67%, p=0.125). Similarly, for the Study-ROIs and the AAL atlases classification remains high at higher regularization. However, in these cases, the best value picked by the algorithm was <5.

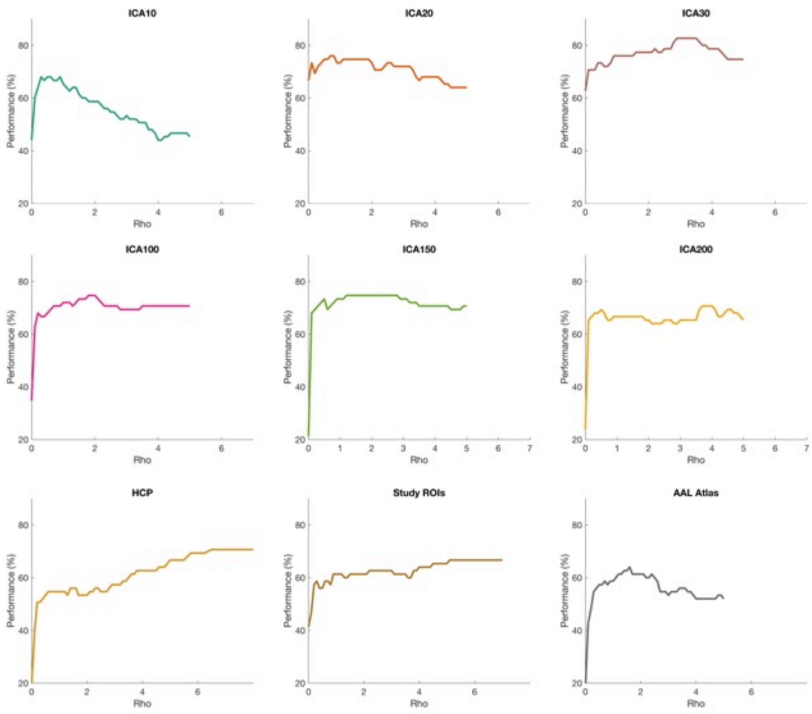

Supplementary Figure 4. Use of different regularization levels and their implication for classification across parcellations. For each parcellation (A-J), we performed sequential classification using a range of regularization values (from 0 to 5, except for HCP and Study ROIs where we tested values from 0 to 8).

Spectral characteristics of Study-ROIs

We studied the power spectra of the regions in the Study-ROIs. For each region, we obtained the mean spectra profile at the different tasks. Besides looking at overall power changes located in low frequencies, we observed small differences in frequencies >0.2 Hz (Supplementary Figures 5 and 6). In addition, we evaluated classification with amplitude and covariance with bandpass filtered data. The results showed good performance of both measures at high-frequencies.

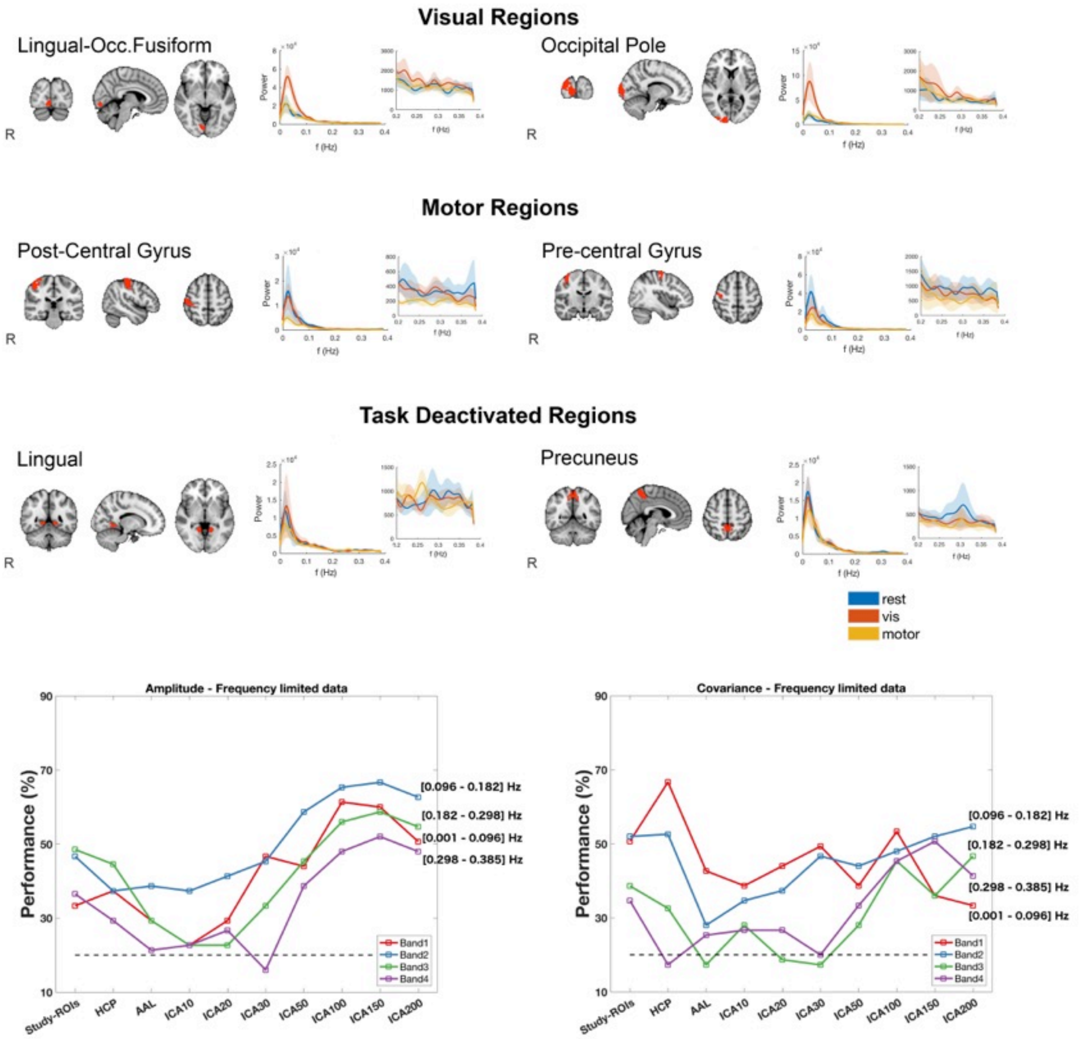

Supplementary Figure 5. Spectral characterization of FC. (A) Spectral profile of selected nodes within the Study-ROIs atlas. For each node, we show its spatial map, the full average spectra and their zoomed spectras at frequencies >0.2 Hz. (B) Classification results using amplitude and covariance of band-limited data.

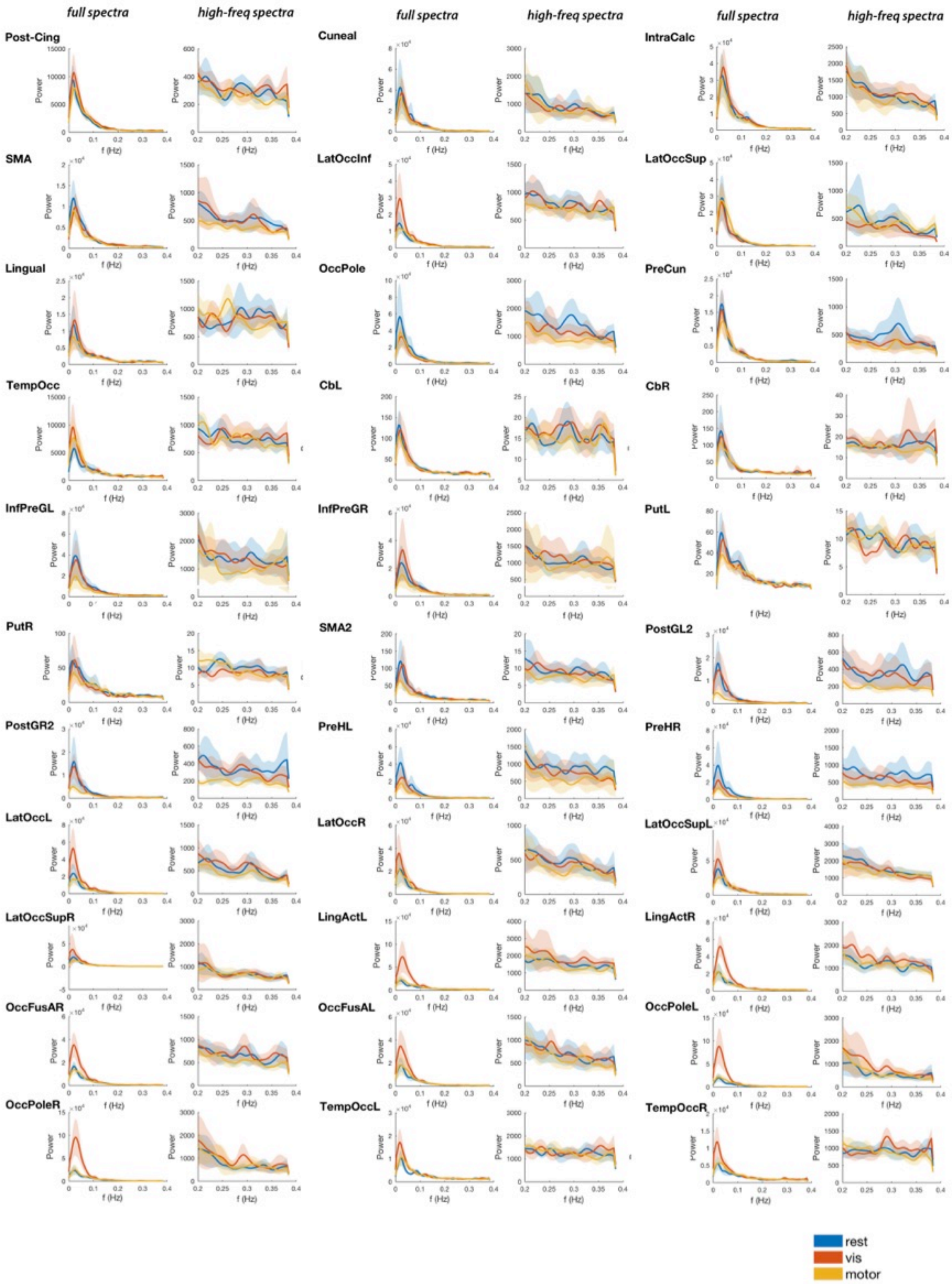

Supplementary Figure 6: Mean spectral profiles for all regions. For each ROI, we show the mean spectra across the full spectra for the resting-state, and the visual and motor states, together with the 'zoomed' spectra showing frequencies above 0.2 Hz. Shadowed areas represent the Confidence Intervals (C.I.)

Testing correlation and partial correlation with band-pass filtered data

We investigated the classification performance of correlation and regularized partial correlation with filtered data. The two lowest bands showed a similar pattern than the one observed with non-filtered data, with good classification results in almost all dimensionalities and with partial correlation often outperforming full correlation. The boost in performance given by the regularized partial correlation was significant for ICA150 in the [0.001 - 0.096] Hz range and for ICA10 and ICA50 in the [0.096 - 0.182] Hz range. In the highest frequency bands, parcellations given by the atlases, the Study ROIs and the lowest ICA decompositions failed to provide good classification rates. However, within these bands, we obtained high classification rates with the finest ICA parcellations. Although the patterns of classification performances were very similar for correlation and partial correlation measures, partial correlation was significantly better in ICA30, ICA50 and ICA150 for the [0.182 – 0.298] Hz range and in AAL, ICA10, ICA30 and ICA200 in the [0.298 – 0.385] Hz range (Supplementary Figure 7).

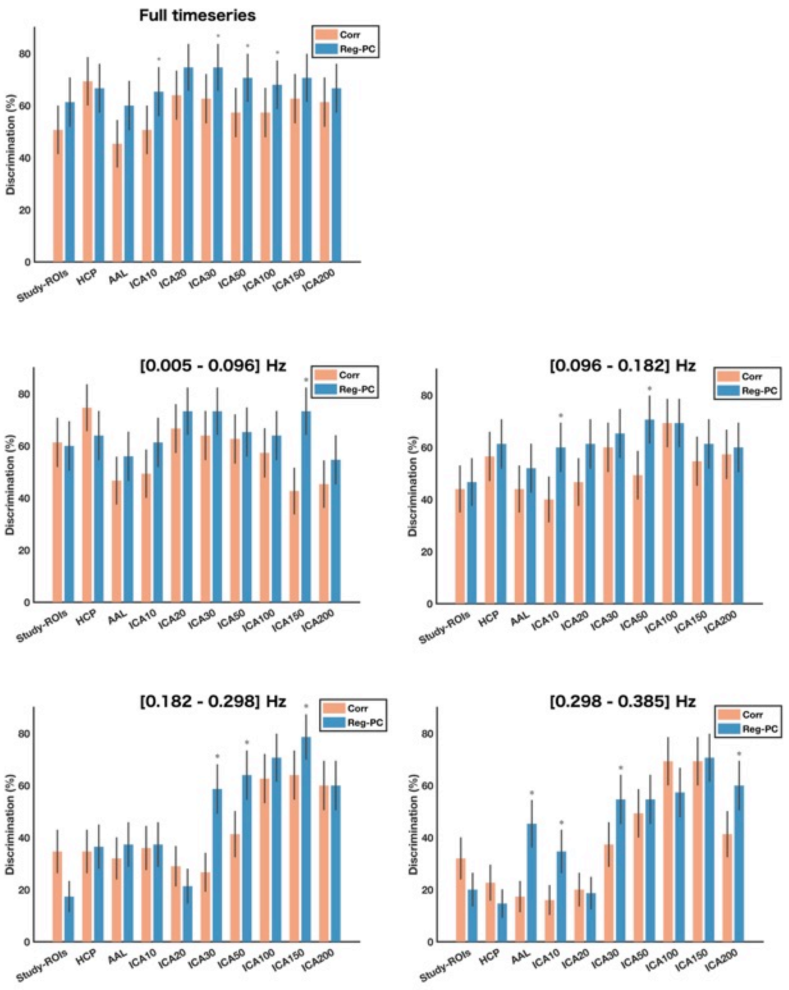

*Supplementary Figure 7. Correlation and partial correlation at different frequency bands. Classification with data filtered at 4 different frequency bands. * indicates that differences between both methods are significant (p<0.05) .*

Additional tests:

We include in this section some additional results that are mentioned but not included in the main manuscript

We first tested classification with other two widely used methods, namely the K-Nearest Neighbor (K-NN) and Random Forest (RF), both implemented in MATLAB. For that, we used the connectivity matrices obtained from the different parcellation schemes and without any additional filtering.

The classification results are similar to those obtained with SVM, being the K-NN slightly lower and the RF less stable than SVM (Supplementary Table IV).

	Study-ROIs	HCP	AAL	ICA10	ICA20	ICA30	ICA50	ICA100	ICA150	ICA200
**K-NN**										
Amp.	46.7%	54.7%	28 %	14.7%	18.7%	29.3%	22.7%	36%	38.7%	45.3%
Cov.	41.3%	49.3%	44%	36 %	37.3 %	48%	40%	58.67%	64%	64.67%
Corr.	65.3 %	58.7%	52%	37.3%	54.7 %	56%	58.7%	70.7%	62.7 %	60%
**RF**										
Amp.	53.3%	49.3%	45.3%	21.3%	29.3%	42.7%	36%	58.7%	62.7%	58.7%
Cov.	58.7%	58.7%	36%	46.7%	61.3%	50.7%	40%	52%	38.7%	45.3%
Corr.	60%	70.7%	46.7%	52%	57.3 %	60%	60%	49.3%	38.7%	34.7%

Supplementary Table IV: Results obtained with alternative classification methods, i.e. K-NN classification and Random-Forest.
K-NN: K-Nearest Neighbor; RF: Random Forest; Amp: Amplitude; Cov: Covariance; Corr: Correlation; Reg-PC: Regularized Partial Correlation

Finally, with the aim to explore whether information from different frequencies was complementary, we performed classification with concatenation of features obtained with the different sets of bandpass filtered data. Even if under some configurations the final classification is a bit higher than the reported for single matrices, in general, we do not observe significant boosts in performance (Supplementary Table V).

Combination	Performance
ICA20 LowFreq. (B1) + ICA150 HighFreq (B3)	78.66%
ICA150 LowFreq. (B1) + ICA150 HighFreq (B3)	81.33 %
ICA10: *concatenate all bands* (B1+B2+B3+B4)	48 %
ICA20: *concatenate all bands* (B1+B2+B3+B4)	57.33 %
ICA30: *concatenate all bands* (B1+B2+B3+B4)	64%
ICA50: *concatenate all bands* (B1+B2+B3+B4)	76 %
ICA100: *concatenate all bands* (B1+B2+B3+B4)	66.67%
ICA150: *concatenate all bands* (B1+B2+B3+B4)	74.67 %
ICA200: *concatenate all bands* (B1+B2+B3+B4)	64%

Supplementary Table V. Classification performance obtained with the combination of connectivity matrices obtained with different bandpass filtering strategies.
B1 = [0.005 - 0.096] Hz; B2 = [0.096 - 0.182] Hz; B3 = [0.182 - 0.298] Hz; B4 = [0.298 - 0.385] Hz
